# AI Awareness and Tobacco Policy Messaging Among US Adults: Electronic Experimental Study

**DOI:** 10.2196/72987

**Published:** 2025-10-27

**Authors:** Julia Mary Alber, David Askay, Anuraj Dhillon, Lauren Sandoval, Sofia Ramos, Katharine Santilena

**Affiliations:** 1 Department of Kinesiology and Public Health, California State Polytechnic University, 1 Grand Ave, San Luis Obispo, CA, 93407, United States, 1 8057561779; 2 Department of Communication Studies, California Polytechnic State University, San Luis Obispo, CA, United States; 3 Department of Biological Sciences, California Polytechnic State University, San Luis Obispo, CA, United States

**Keywords:** artificial intelligence, tobacco control, health communication, generative artificial intelligence, health policies

## Abstract

**Background:**

Despite public health efforts, tobacco use remains the leading cause of preventable death in the United States and continues to disproportionately affect underrepresented populations. Public policies are needed to improve health equity in tobacco-related health outcomes. One strategy for promoting public support for these policies is through health messaging. Improvements in artificial intelligence (AI) technology offer new opportunities to create tailored policy messages quickly; however, there is limited research on how the public might perceive the use of AI for public health messages.

**Objective:**

This study aimed to examine how knowledge of AI use impacts perceptions of a tobacco control policy video.

**Methods:**

A national sample of US adults (N=500) was shown the same AI-generated video that focused on a tobacco control policy. Participants were then randomly assigned to 1 of 4 conditions where they were (1) told the narrator of the video was AI, (2) told the narrator of the video was human, (3) told it was unknown whether the narrator was AI or human, or (4) not provided any information about the narrator.

**Results:**

Perceived video rating, effectiveness, and credibility did not significantly differ among the conditions. However, the mean speaker rating was significantly higher (*P*=.001) when participants were told the narrator of the health message was human (mean 3.65, SD 0.91) compared to the other conditions. Notably, positive attitudes toward AI were highest among those not provided information about the narrator; however, this difference was not statistically significant (mean 3.04, SD 0.90).

**Conclusions:**

Results suggest that AI may impact perceptions of the speaker of a video; however, more research is needed to understand if these impacts would occur over time and after multiple exposures to content. Further qualitative research may help explain why potential differences may have occurred in speaker ratings. Public health professionals and researchers should further consider the ethics and cost-effectiveness of using AI for health messaging.

## Introduction

Despite some progress in tobacco control and prevention, tobacco remains the leading cause of preventable death in the United States [1], with persistent inequities in health outcomes among youth, rural residents, people with mental health illnesses, communities of color, and the Lesbian, Gay, Bisexual, Transgender, Queer or Questioning, Intersex, Asexual + community [2,3]. Secondhand smoke exposure disproportionately impacts Black people compared to any other racial group, putting them at higher risk for asthma and long-term lung disease [2,4]. Public policies are essential for addressing health inequities associated with tobacco use and exposure [2].

While there is evidence that policies can decrease smoking behaviors [5], there is a need to implement more comprehensive policies to promote and achieve health equity in the United States. Given this need, more countries and US jurisdictions are moving toward “Endgame” policies [6]. Endgame policies focus on strategies that address existing health inequities and target commercial sales of tobacco products rather than individual use. Existing research in New Zealand indicates some support for Endgame policies [7-9]. For example, flavored tobacco products have been specifically targeted to communities of color and youth, which in turn has led to higher tobacco-related disease and death among these communities [10-13]. One potential Endgame strategy to reduce these inequities is a policy banning the sale of tobacco products.

While research has examined the overall effectiveness of some tobacco control policies, there is limited research on how to best promote support for these types of policies. Developing and testing messaging for promoting tobacco Endgame policies is essential for promoting local, state, and national efforts in the United States to address tobacco-related inequities. However, developing and testing high-quality content, particularly video content, can be expensive and time-consuming, given that it can require a significant investment of resources [14].

With limited resources, public health organizations such as local tobacco control coalitions often lack the time, funding, or expertise to design high-quality content. Generative artificial intelligence (AI) offers tools to address these challenges by enabling the rapid creation of targeted messages, supporting simple analyses of communication features (eg, pause counts), and producing high-quality videos [15-18]. Platforms such as ChatGPT (OpenAI) can generate scripts or social media text within seconds, while other programs can create videos featuring digital avatars that deliver messages with customizable voices and appearances. These capabilities can reduce costs, shorten production time, and allow messages to be tailored to specific audiences. For example, one study found that AI-generated health messages were rated higher in quality and clarity than human-generated messages from Twitter (xAI; [19]), although the findings were limited to a sample of college and young adult women.

With this in mind, AI messages can help tailor messages to a specific population. In addition, the use of AI, such as ChatGPT, can be used to decrease language barriers in health care and simplify medical jargon to make it more accessible [20]. Incorporating AI in health messaging has been associated with several positive outcomes, including increased clarity, improved message tailoring, and reduced language barriers in health communication [19,20]. Within tobacco control, AI is already being used to personalize quitting interventions (eg, chatbots for cessation support) and to monitor tobacco-related trends (eg, social media surveillance) [21].

Overall, AI has the potential to revolutionize health communication and allow public health organizations to create quality content more quickly and cost-effectively. However, understanding how the public perceives the use of AI for health messaging is important. Research highlights several barriers to accepting messages involving AI, including concerns that such messages may be perceived as less trustworthy or relatable, and discomfort associated with AI entities mimicking human characteristics (commonly referred to as the “uncanny valley”) [22]. For example, though traditionally applied to visual AI, the “uncanny valley” may also occur with synthetic voices that mimic human voices but may lack natural emotion or fluctuation, potentially eliciting audience discomfort [22]. These factors can diminish the perceived effectiveness and credibility of public health messages.

Existing theoretical frameworks can help explain how audiences perceive AI in health messaging. The elaboration likelihood model (ELM) describes 2 routes of information processing: the central and peripheral [23]. When individuals have little interest in or connection to a topic, they are less likely to critically think about the information presented [23] and instead rely on heuristics such as source credibility to make decisions [23]. For example, the source or speaker in a message and their perceived credibility could influence individuals’ decisions to follow or not follow the message. In the case of AI-delivered messages, positive perceptions of AI may increase acceptance of the message, whereas negative perceptions of AI may lead audiences to discount or ignore it.

The computers as social actors (CASA) paradigm further shows that people apply social expectations such as trust and empathy to digital agents [24]. Recent work has extended CASA to health communication contexts, demonstrating that perceived characteristics of AI sources can shape message credibility and audience engagement [25]. In health messages, when trust and credibility are vital to the processing of a message, audiences may evaluate AI speakers as more or less credible than human ones, even when delivering identical content. These frameworks highlight key factors shaping how audiences interpret and respond to AI-delivered health information.

To the authors’ knowledge, no existing research has specifically investigated how perceptions of an AI-generated speaker may impact message outcomes in an experimental design using both qualitative and quantitative data. Therefore, the purpose of this study was to explore whether awareness that a speaker is AI-generated influences the message outcomes of a tobacco policy video. We hypothesized that participants who were informed that the narrator was AI-generated would rate the video lower in overall quality, perceived effectiveness, credibility, and speaker evaluations compared with those who were told that the narrator was human, unknown, or not informed.

## Methods

### Recruitment and Data Collection

Data were collected between January 25 and February 1, 2024. US adults (N=500) were recruited through the Qualtrics Survey Panel for an electronic survey. Quota sampling was used to approximate the US adult population distribution for age, sex, race, and ethnicity based on the American Community Survey from the US Census Bureau [26]. A priori power analysis using G*Power (Erdfelder, Faul, and Buchner) indicated that a sample of this size was sufficient to detect a small effect (*f*=0.15) with 80% power at *α*=.05 in a 4-condition ANOVA.

Participants were recruited through an electronic survey panel (Qualtrics Panel) that maintains a large pool of individuals who voluntarily register to participate in research. Qualtrics Panels use a standard recruitment email message that includes information on compensation, time to complete, and an anonymous survey link, which is sent to the registered pool of participants. Participants who meet eligibility criteria, with quotas applied to match US Census benchmarks for age, sex, and race and ethnicity, are profiled and invited to studies. Participants are compensated for participation according to the panel’s standard compensation options, which can vary based on participation. The panel company managed recruitment and provided researchers with the final sample. The Qualtrics Panel has been used to collect data for other studies examining health beliefs and message testing [27-29].

**Figure 1.**
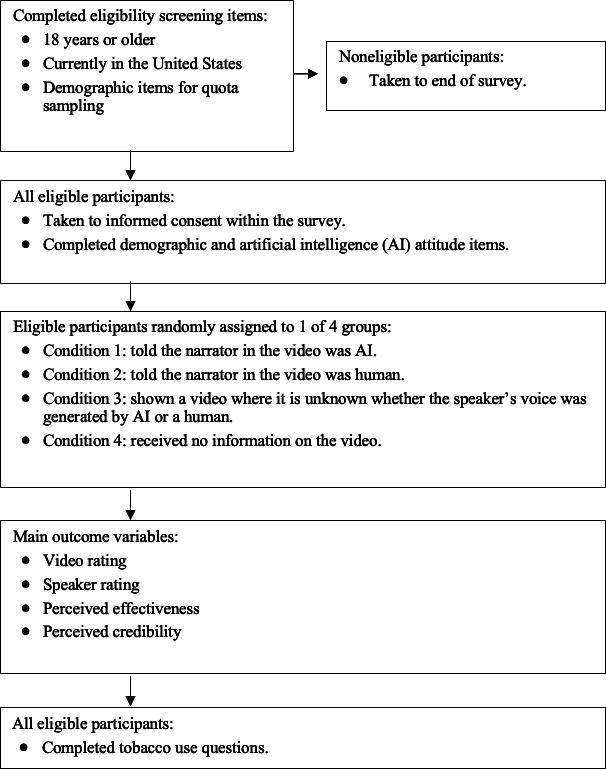
Overview of study procedures for a randomized electronic survey assessing perceptions of an artificial intelligence (AI)-narrated tobacco control video among US adults, 2024.

The procedures for the survey are summarized in Figure 1. Within the survey, each participant was randomly assigned to 1 of 4 conditions. First, participants were exposed to a video narrated by an AI developed using PlayHT (voice-generating software; Mahmoud Felfel and Hammad Syed). The video depicted a narrator telling a story about how their addiction to flavored vapes started and how this addiction controlled their life. At the end of the video, a message promoting the end of flavored tobacco sales was displayed. The only thing participants saw in the video was the words being spoken and a consistent background. Participants were then presented with different text information from 1 of 4 conditions: (1) told the narrator in the video was AI, (2) told the narrator in the video was human, (3) told it was unknown whether the narrator’s voice was generated by AI or a human, and (4) given no mention of the narrator’s source, allowing participants to experience the video naturally without bias or preconceived notions about its creation. Despite participants being informed that the narrator’s voice was human, AI-generated, unknown, or not mentioned, deception was used. The same video featuring an identical AI-narrator voice was used across all conditions. This strategy ensured consistency, minimized variability, and allowed the researchers to focus exclusively on the effects of the informational context provided to participants. Several strategies were used to identify inattentive or fraudulent participants, including attention-check measures, checking the accuracy of open-ended responses, and speed checks (Multimedia Appendix 1).

### Measures

Demographic variables were used to collect information on participants’ sex, race, ethnicity, marital status, income, and education, as well as their tobacco use, based on established surveys [1,28]. In addition, AI attitudes were assessed by having participants rate their agreement from 1 (strongly disagree) to 5 (strongly agree) for 8 statements (eg, “I think artificial intelligence is dangerous”) [30]. An average was then calculated for positive and negative AI attitudes (Cronbach α=0.79 and 0.71, respectively). Four main message outcome measures were used: video rating, perceived effectiveness, perceived credibility, and speaker rating.

Video rating was measured by asking participants how much they liked or disliked the video on a scale from 1 (like a great deal) and 7 (dislike a great deal) [31]. To measure perceived effectiveness, participants rated their agreement from 1 (strongly disagree) to 5 (strongly agree) for 6 statements about the video being convincing, compelling, persuasive, effective, saying something important to them, and putting thoughts in their mind about supporting a flavored tobacco sales ban. The items were developed based on previous research (Cronbach α=0.91) [31,32]. Perceived credibility (Cronbach α=0.89) was assessed by asking participants to rate their level of agreement from 1 (strongly disagree) to 5 (strongly agree) that the information was accurate, believable, and factual [33]. To measure speaker rating (Cronbach α=0.97), participants rated the person speaking in the video from 1 (not at all) to 5 (extremely) on 20 characteristics (bright, cares about me, competent, concerned with me, credible, ethical, expert, genuine, has my interest at heart, honorable, informed, intelligent, knowledgeable, likable, moral, not self-centered, sensitive, trained, trustworthy, and understanding). These items were adapted from previous research [28,34,35]. Scores for speaker rating, perceived effectiveness, and perceived credibility were created by averaging across the items in each scale.

### Statistical Analysis

SPSS (version 29; IBM Corp) was used to examine frequency and central tendency measures of demographic characteristics and tobacco use variables. To check randomization efficacy, chi-square and 1-way ANOVA tests were used to examine if any condition differences existed among participant characteristics. One-way ANOVAs were used to examine if condition differences existed among video rating, perceived effectiveness, perceived credibility, and speaker rating. In addition to ANOVA statistical tests, linear regression analyses were conducted to determine whether conditions predicted message outcomes (ie, speaker rating, perceived effectiveness, and credibility) while controlling for variables (ie, age, gender, Black or African American, Latinx, education, marital status, flavored product use, lifetime cigarette use, and AI attitudes). Dummy coding was used for the regression analysis, with the AI-narrated condition (Condition 1) set as the reference group, allowing comparisons between this condition and each of the other conditions.

### Qualitative Analysis

A thematic analysis was performed to categorize participants’ written comments for each video [36]. The process involved 2 trained research assistants creating initial codes using a sample of comments, defining codes, pilot testing the codes until acceptable intercoder reliability was established (≥90% agreement), coding the remaining data, and grouping the codes into themes. Only codes representing at least 5% of participants were included in the themes. Theme frequency was then explored by participant conditions using NVivo Qualitative Data Analysis Software (version 16; Lumivero).

### Ethical Considerations

Before data collection, California Polytechnic State University’s Institutional Review Board approval was obtained (approval #2023‐223-OL). All participants provided informed consent electronically before participating in the study. Data were collected anonymously and participants were compensated according to their survey panel’s normal compensation options.

## Results

### Demographic Characteristics

Demographic information is presented in Table 1. Participants were, on average, the age of 48.42 (SD 17.66) years, with an equal split identifying as male (n=245, 49%) and female (n=250, 50%). The majority of participants identified as White (n=385, 77.0%), while approximately 48.7% (n=215) of participants reported making less than US $50,000 annually. The majority of participants had more than a high school education (n=398, 79.8%).

In terms of tobacco use, 175 (35%) participants reported having smoked at least 100 cigarettes in their lifetime, and 146 (29.2%) participants reported ever using flavored tobacco products.

**Table 1.** Demographic characteristics, tobacco use, and message outcome measures among US adults (N=500) participating in an electronic survey on artificial intelligence (AI)–narrated tobacco control messaging, United States, 2024.

Characteristic or outcome variables		Condition 1: told video is AI (n=116)^a^	Condition 2: told video is human (n=138)	Condition 3: told video is unknown (n=121)	Condition 4: no additional information (n=125)	Difference by condition, *P* value
Age (years), mean (SD)		47.93 (17.24)	49.92 (18.91)	49.02 (17.7)	46.65 (17.67)	.48
Sex, n (%)						.50
Male		63 (54.3)	63 (45.7)	61 (50.4)	58 (46.4)	
Female		52 (44.8)	74 (53.6)	58 (47.9)	66 (52.8)	
Income, n (%)						.44
<US $25,000/year		19 (16.7)	30 (21.9)	29 (24.2)	30 (24.6)	
≥US $25,000/year		95 (83.3)	107 (78.1)	91 (75.8)	92 (75.4)	
Race, n (%)						
White		91 (78.4)	106 (76.8)	97 (80.2)	91 (72.8)	.56
Black		15 (12.9)	17 (12.3)	16 (13.2)	16 (12.8)	.10
American Indian or Alaska Native		2 (1.7)	2 (1.4)	1 (0.8)	3 (2.4)	.80
Asian		6 (5.2)	10 (7.2)	4 (3.3)	9 (7.2)	.49
Pacific Islander		1 (0.9)	0 (0)	1 (0.8)	0 (0)	.53
Other		4 (3.4)	6 (4.3)	3 (2.5)	9 (7.2)	.30
Latinx		12 (10.3)	28 (20.3)	22 (18.2)	28 (22.4)	.08
Education, n (%)						.57
High school or below		20 (17.2)	29 (21.2)	29 (24.0)	23 (18.4)	
Above high school		96 (82.8)	108 (78.8)	92 (76.0)	102 (81.6)	
Marital status, n (%)						.79
Married		48 (41.4)	56 (40.6)	56 (46.3)	55 (44.0)	
Other		68 (58.6)	82 (59.4)	65 (53.7)	70 (56.0)	
Flavored product use among ever-users, n (%)						.99
Everyday or someday		16 (45.7)	20 (43.5)	15 (46.9)	16 (45.7)	
Not at all or do not know		19 (54.3)	26 (56.5)	17 (53.1)	19 (54.3)	
Lifetime cigarette use, n (%)						.14
Yes		33 (28.9)	56 (42.4)	46 (38.0)	40 (33.1)	
No		81 (71.1)	76 (57.6)	75 (62.0)	81 (66.9)	
Video rating, mean (SD)		3.22 (1.62)	2.73 (1.53)	3.08 (1.64)	2.82 (1.68)	.07
Perceived effectiveness, mean (SD)		3.51 (1.02)	3.75 (1.01)	3.71 (0.98)	3.79 (0.10)	.14
Message credibility, mean (SD)		4.03 (0.93)	4.20 (0.81)	4.07 (0.87)	4.19 (0.86)	.29
Speaker rating, mean (SD)		3.25 (0.93)	3.65 (0.91)	3.35 (0.99)	3.60 (0.96)	.001^b^
Positive AI attitudes, mean (SD)		2.84 (0.88)	2.93 (0.88)	2.82 (0.79)	3.04 (0.90)	.17
Negative AI attitudes, mean (SD)		2.44 (0.87)	2.49 (0.88)	2.44 (0.86)	2.67 (0.90)	.15

a AI: artificial intelligence.

b Statistically significant.

### Message Outcomes

Speaker ratings showed a significant difference across conditions (*P*=.001), with the highest ratings observed in Condition 2 (told the narrator was human; mean 3.65, SD 0.91). Condition 2 (told the narrator human; mean 3.65, SD 0.91) had significantly higher speaker rating compared to Condition 1 (told the narrator was AI; mean 3.25, SD 0.93). Condition 4 (no additional information provided; mean 3.60, SD 0.96) also had a significantly higher speaker rating compared to Condition 1 (told the narrator was AI; mean 3.25, SD 0.93). In contrast, there were no significant differences across conditions in video rating, perceived effectiveness, or perceived credibility. While not statistically significant, the largest difference in video rating was between Condition 1 (told the narrator was AI; mean 3.22, SD 1.62) and Condition 2 (told the narrator was human; mean 2.73, SD 1.53). Condition 1 also showed the lowest perceived effectiveness (mean 3.51, SD 1.02). Interestingly, positive AI attitudes were highest in Condition 4 (no information provided about the narrator; mean 3.04, SD 0.90), though this difference was also not statistically significant.

A linear regression analysis revealed that being told the narrator was human was significantly associated with higher speaker ratings compared to being told the narrator was AI (B=0.69; *P*=.003; F_13,125_=2.78; *P*=.002). Similarly, positive AI attitudes were also associated with higher speaker ratings (B=0.40; *P*<.001). For perceived effectiveness, there were no significant differences between conditions. However, participants with more positive attitudes toward AI were more likely to rate the message as effective (B=0.43; *P*<.001). Regression models for video rating and message credibility outcomes were not significant.

### Qualitative Comments

The results from the qualitative comment analysis produced 4 themes, including Vaping Outcomes, Video Negative Content, Voice, and Video Positive Content. The themes, theme definitions, corresponding codes, code frequencies within the dialog, and examples of corresponding quotes are outlined in Table 2. There were some notable differences and similarities in the qualitative comments across conditions.

**Table 2.** Thematic analysis of participant responses to a tobacco control video narrated by artificial intelligence (AI): summary of themes, definitions, and example codes from a randomized electronic survey of US adults, 2024.

Themes and their codes	Representative quote
Vaping Outcome	
Participants described their beliefs or attitudes toward tobacco products and vaping in general. This includes their views on the addictive qualities, financial costs, or health implications of these substances, as well as general criticisms and negative perceptions.	
Health	“Vaping ruins your health”
Cost	“...vaping is expensive.”
Addiction	“...the thought was the importance of someone choosing a drug over enjoying a good time is a sad case of addiction”
Vaping negative	“People should listen, vaping is dangerous and addictive, don’t believe commercials about vaping not being addictive.”
Video Negative Content	
Participants provided negative feedback on the video related to its credibility, engagement, persuasiveness, or personal relevance. This feedback also involved comments about possible improvements to the video and what the video lacked.	
No factual	“The ad did not use fact or science to back up its point.”
Boring	“It didn’t apply to me or anything I would find useful and was actually pretty boring.”
Disagreement	“It could help someone who is thinking of quitting… but it also just makes me feel more determined that having a ban on vape flavors is not the answer…”
Not convincing	“I thought it was sounding more like a novel, a fictitious one at that. I also thought that if the advertisement was trying to convince someone not to vape, then they failed because there was no sign of it having anybody’s best interests in mind. It sounds like they were reading a story.”
Not applicable	“It was targeted at adults. This is more of a youth or teenager issue in my opinion.”
Voice	
Participants mentioned the narrator’s voice being AI^a^ or sounding monotone.	
AI detected	“... you could tell it was AI spoken”
Monotone	“The voice is monotone and dead. Creepy.”
Video Content Positive	
Participants described the content of the video in a positive way. This included how well the ad conveys its message, connects with viewers on a personal level, and motivates action or awareness.	
Factual	“…gives facts”
Take action	“People need to be more informed about the dangers of vaping.”
Compassion	“...this person lost everything, spent thousands, and damaged her lungs.”
Personal	“It was very powerful. My husband died as a result of his nicotine addiction. This message hits home.”
Convincing	“The ad was convincing to me, explaining the results of vaping addiction.”
Genuine	“...is informative, genuine, vulnerable, and authentic”
Informative	“This video is very Informative, true, and inspiring.”
Positive	“It was empowering.”

a AI: artificial intelligence.

For Condition 1 (participants told the video was AI), the most commonly referenced code was “informative” (n=21). This was closely followed by the perception of the theme “voice” being “monotoned,” referenced 20 times, and the theme “vaping outcomes” coded as “addictive,” referenced 19 times within Condition 1’s dialog.

Participants who were told the narrator in the video was human (Condition 2) most commonly referenced with the code “vaping negative” within the theme “vaping outcome” (n=36). The second most referenced code within the condition was “positive” within the theme “video content,” referenced 29 times.

In Condition 3 (participants told it was unknown whether the narrator was human or AI), the most recurring codes were within the theme “video content,” coded as “informative” and “positive,” and the theme “vaping outcomes,” which produced the code “negative outcomes,” both referenced 25 times in the comments. This was followed closely by the theme “video content” being perceived as “convincing” and “positive,” occurring 22 times.

Similar to Condition 2, Condition 4 (participants were given no information about the narrator) most commonly included the code “vaping negative” within the theme “vaping outcome,” referenced 46 times within that condition. The second most recurring code in Condition 4 was “positive” within the theme “video content,” referenced 36 times. This result was similar to Condition 2, where the highest recurring codes were “Theme: vaping outcome, code: vaping negative” and “Theme: video content, code: positive.”

Across the conditions, the most frequent was “vaping negative” within the theme “vaping outcome,” referenced 12 times in Condition 1, 36 times in Condition 2, 25 times in Condition 3, and 46 times in Condition 4. Another common code across conditions was “informative” within the theme “video content positive” (Table 3).

**Table 3.** Frequency of qualitative themes coded from open-ended responses by experimental condition (narrator artificial intelligence [AI] identity disclosure).

Themes and their codes	Condition 1: told video is AI^a^ (n=116)	Condition 2: told video is human (n=138)	Condition 3: told video is unknown (n=121)	Condition 4: no additional information (n=125)
Video Content Positive, n (%)				
Informative	21 (18)	21 (15)	25 (20)	21 (17)
Compassion	15 (13)	12 (8)	9 (7)	8 (6)
Positive	15 (13)	29 (21)	22 (18)	33 (26)
Personal	14 (12)	17 (12)	15 (12)	9 (7)
Genuine	10 (8)	14 (10)	22 (18)	18 (14)
Convincing	9 (7)	14 (10)	22 (18)	11 (9)
Take action	8 (6)	7 (5)	9 (7)	8 (6)
Factual	6 (5)	10 (7)	8 (6)	5 (4)
Voice, n (%)				
Monotone	20 (17)	11 (8)	17 (14)	14 (11)
AI detected	14 (12)	4 (3)	7 (5)	3 (2)
Real voice	4 (3)	1 (0.7)	3 (2)	4 (3)
Vaping outcome, n (%)				
Addictive	19 (16)	20 (14)	16 (13)	29 (23)
Negative	12 (10)	36 (26)	25 (20)	46 (37)
Cost	10 (8)	13 (9)	13 (10)	17 (13)
Health	7 (6)	8 (5)	4 (3)	8 (6)
Video content negative, n (%)				
Not applicable	9 (7)	12 (8)	11 (9)	6 (4)
Not convincing	9 (7)	5 (3)	11 (9)	7 (5)
Not factual	9 (7)	6 (4)	5 (4)	7 (5)
Boring	5 (4)	6 (4)	7 (5)	9 (7)
Disagreement	5 (4)	6 (4)	8 (6)	7 (5)

a AI: artificial intelligence.

## Discussion

### Principal Findings

This study explored how perceptions of narrator identity (AI vs human) influence audience evaluations of a health messaging video focused on a tobacco control policy. It was hypothesized that participants informed that the narrator was AI-generated would rate the video lower on video rating, perceived effectiveness, credibility, and speaker ratings compared to those told the narrator was human, unknown, or not mentioned. Importantly, across all conditions, the narration was AI-generated, even when participants were told that it was human. In addition, the study found similarities and differences in qualitative feedback that differed by condition.

The findings partially supported the hypothesis. Quantitative results revealed that speaker ratings were significantly higher when participants were told the narrator was human, suggesting that perceived narrator identity may change perceptions toward the narrator, even when the narration itself is identical. The significant differences in speaker ratings, despite the identical message, suggest that source cues like whether a narrator is perceived as AI may shape how individuals evaluate a speaker. These findings indicate that perceptions of the narrator can influence speaker evaluations, though the quality of the content itself may help mitigate some of these biases. Trends showing slightly lower ratings for videos where the narrator was described as AI further align with existing research that highlights biases against AI-driven communication in trust-sensitive contexts, such as health messaging [37].

Qualitative findings added depth to the results of this study by illustrating how perceptions of narrator identity shaped audience engagement. Participants in the AI-narrator condition (Condition 1) frequently described the voice as “monotone” or “unnatural,” potentially detracting from the video’s emotional resonance. In contrast, participants in the human-narrator condition (Condition 2) described the same content as “genuine,” “convincing,” and “relatable.” This suggests that perceived authenticity and relatability are critical to audience engagement. Interestingly, participants in the no-information condition (Condition 4) focused primarily on the video’s content, referencing its informative nature and the health impacts of vaping. Across all conditions, participants consistently highlighted the video’s “informative” content, underscoring the importance of factual accuracy and relevance in health messaging.

In addition, participants’ overall attitudes toward AI did not show any significant difference among the conditions. This may suggest that overall perceptions of AI may be less influential than specific perceptions toward the narrator of a message. This study offers a novel contribution by examining how knowledge of an AI narrator may influence perceptions of a tobacco control policy message, using a controlled design where the message content remains constant across conditions. Unlike previous research that analyzed existing human-generated content (ie, Twitter posts), this design isolates the effect of the perception of the speaker by removing confounding factors (eg, message length and quality of the message) [19]. In addition, the inclusion of qualitative data, which is currently scarce in AI message research, provides deeper insight into audience reactions to AI-generated speakers. Taken together, these findings suggest that public health professionals should prioritize shaping perceptions of the speaker as a central factor in developing effective AI-based health communication strategies, rather than focusing solely on broad attitudes toward AI.

### Limitations

There are several limitations to note that should be addressed in future research. Participants were only exposed to one video, which may not have provided sufficient opportunity to fully process and evaluate the content. In addition, while participants were told the narrator was AI or human, all conditions featured the same AI-generated voice. This approach controlled for variability but did not allow for direct comparison with an actual human-narrated video. Future studies should include both human and AI narrators to better understand audience preferences and perceptions.

### Comparison With Prior Work

Other research has shown that negative attitudes toward AI may influence message perceptions. In one study comparing the evaluation of AI versus human-generated messages, researchers found moderating effects of negative attitudes toward AI on the evaluation of the messages, but not on which message was ultimately selected [20]. Given the limitations of this study and existing research in terms of study design, exposure, and lack of measurement over time, more research is needed to better understand the role of general AI attitudes in message outcomes.

While not significant, there was a trend in the overall video rating that was similar. With the current findings, there is some indication of preferences toward using human-generated videos compared to AI. Other studies have found that there may be mistrust and suspicion regarding AI involvement in health content, as it is a newer generative digital media tool [37]. As AI continues to evolve and update, AI-related concerns persist and could contribute to the study’s result of lower ratings among the videos where participants were told there was an AI narrator. In a recent study comparing human-made and AI-generated teaching videos, researchers found similar results of participant preferences for human-made videos. However, results between conditions demonstrated participants acquired the same level of knowledge whether the video was human-made or not [38]. Another study examining the effectiveness of health messages, specifically related to COVID-19 vaccination, between human and nonhuman sources found participants perceived the message as more credible and more influential if the message came from a human in comparison to an AI communicator [39]. These findings are similar to the results of this study, finding a preference for human interaction over AI, but demonstrate the potential of AI-generated materials as an adequate means of effectively portraying information for an intended audience.

In the context of tobacco control policies, this research provides some evidence that generative AI could be used to develop messages to promote policy support. Although differences were observed in speaker ratings, other outcomes, such as video likability and perceived effectiveness, were not significant. Previous work on tobacco prevention campaigns has emphasized the importance of tailoring narratives to resonate with specific communities [7,8]. Generative AI offers potential for such tailoring, particularly for communities disproportionately affected by flavored tobacco [16]. In addition, it has the potential to be used to counteract misinformation and pro-tobacco content on social media [40]. In a recent study, researchers compared generative AI messages to existing organizational messages for vaping prevention [41]. Contrary to our current findings, AI-generated messages outperformed the existing messages. It is important to note, however, that this previous work compared different messages, while this study used the same video across conditions.

While this study did not find differences in perceived credibility among conditions, researchers and practitioners should remain attentive to the accuracy of generative AI output [42]. For example, one content analysis found that ChatGPT-generated content for smoking cessation was accurate only 57% of the time [43]. Given the lack of published research studying the effectiveness and cost-effectiveness of using generative AI for promoting tobacco control policies, more research is needed to understand how this technology can be leveraged most effectively by public health professionals.

### Implications for Practice

The findings highlight important considerations for public health practitioners seeking to integrate AI into health messaging campaigns. One key takeaway is the significant influence of perceived narrator identity on speaker ratings. Participants rated the narrator higher when they believed it to be human, even though the narration was AI-generated in all conditions. This underscores the need to address biases against AI in contexts where trust and emotional engagement are critical. However, it was noted that general AI attitudes were not different across the conditions. Strategies such as framing AI as a collaborative tool or emphasizing human oversight in content creation may help mitigate these biases. For example, campaigns could position AI as enhancing efficiency and personalization while maintaining human involvement to reassure audiences.

The qualitative results emphasize the importance of emotionally engaging and factually accurate content. Participants across conditions frequently described the video as “informative,” indicating that high-quality messaging can resonate with audiences, regardless of the narrator’s perceived identity. This suggests that the strength of the content itself can help overcome biases against AI-generated communication. Public health practitioners should prioritize creating clear, relevant, and accurate messages that address audience needs and expectations.

Finally, findings from the no-information condition (Condition 4) suggest that omitting explicit mention of AI involvement can help audiences engage more neutrally with the content. When ethical considerations allow, avoiding disclosure of the narrator’s source may reduce bias and encourage audiences to focus on the message itself. However, transparency remains essential in some contexts, and practitioners must carefully balance trust-building with strategies to mitigate audience biases.

Overall, these insights highlight the potential for AI to serve as a valuable tool in health messaging. Its cost-effectiveness, scalability, and ability to tailor content for diverse populations may make it a particularly useful resource for organizations with limited budgets or technical expertise [38]. However, more research is needed to fully understand the feasibility and cost-effectiveness of using AI within public health organizations. By addressing biases and emphasizing content quality, practitioners can leverage AI to deliver impactful, audience-centered health messages.

### Implications for Theory

This study contributes to the theoretical understanding of audience responses to AI in health communication. The significant preference for human narrators in speaker ratings reinforces existing theories that emphasize the importance of trust and relatability in communication [39]. These findings align with the Social Presence Theory [44], which suggests that perceptions of the communicator’s presence—whether real or artificial—can significantly impact how messages are received. In this study, even though the content and delivery were identical, participants’ evaluations were shaped by their perceptions of narrator identity, demonstrating that trust and relatability remain pivotal in audience engagement. The results also somewhat align with ELM. It appeared that the “cue” of knowing whether the speaker was AI or not influenced how participants perceived the speaker and processed the information in terms of intention to follow the recommendations of the video. Interestingly, using the CASA paradigm, knowledge of AI did not appear to impact trust or perceived credibility in the video.

The qualitative findings reveal how perceptions, rather than actual differences in narration, drive audience engagement. Participants who believed the narrator was human described the content as “genuine” and emotionally resonant, while those who told the narrator was AI frequently noted a lack of authenticity. This underscores the critical role of perceived authenticity in shaping trust and engagement, which is supported by the Heuristic-Systematic Model [45]. According to this model, individuals often rely on heuristics, such as perceived human authenticity, to evaluate messages when systematic processing is not fully engaged. The findings reinforce the importance of narrative framing in health communication, as even subtle cues about the narrator’s identity can influence audience perceptions and ultimately the effectiveness of the message.

Interestingly, the lack of significant differences in video rating, perceived effectiveness, and credibility across conditions suggests that high-quality content can mitigate some biases against AI. This finding underscores the importance of focusing on the intrinsic value of the message itself—clarity, relevance, and informativeness—over the medium or delivery method. Theoretical models, such as the ELM [23], emphasize that when the content of a message is highly relevant and cognitively engaging, audiences are more likely to process the message through the central route, leading to deeper understanding and less susceptibility to peripheral biases, such as those linked to the narrator’s identity. Similarly, research on health communication has shown that message quality and perceived relevance are among the strongest predictors of message effectiveness, even in digital or mediated contexts [5]. These findings suggest that public health campaigns leveraging AI should prioritize delivering clear and relevant information to ensure audience engagement and mitigate potential biases.

### Future Research

This study fills a gap in research examining reactions to AI-involved health messaging videos. Future research could build on these findings to examine whether message outcomes differ over time with multiple exposures to different types of videos. In addition, studies could compare different AI visuals, such as avatars, graphics, or cartoons, to determine likability across AI technologies. Examining emotional reactions or features of AI-generated emotions is also needed. Finally, examining the usability and cost-effectiveness of AI within public health settings would better inform the overall utility of using it for health messaging. This study did not examine the cost-effectiveness; however, some research shows that AI can be a cost-effective and rapid means of producing content, which is a common reason more industries use it in outreach materials [37]. It would be important to determine the cost-effectiveness and usability of AI within public health settings where resources and training may be limited. Overall, AI is a tool with great potential in future health messaging and outreach, but obstacles remain related to the intended population’s attitude, trust, and future acceptance.

### Conclusions

As AI continues to evolve and programs become more human-like, more organizations are adapting to use AI. This study found that speaker ratings were significantly influenced by the perceived identity of the narrator, with participants rating the speaker significantly higher when they believed the voice was human. However, no significant differences were observed for perceived effectiveness, credibility, or overall video ratings across conditions. These findings suggest that message sources, particularly regarding AI, can influence an audience’s perceptions of the speaker even when content remains constant.

## Supplementary material

10.2196/72987 Multimedia Appendix 1 Link to the video shown to participants.

## Abbreviations

AI: artificial intelligence
CASA: computers as social actors
ELM: elaboration likelihood model
