# Supplementary material for: AI Awareness and Tobacco Policy Messaging Among US Adults: Electronic Experimental Study
Source: JMIR AI. 2025 Oct 27;4:e72987. doi: 10.2196/72987 (PMC12558419; doi:10.2196/72987)
Supplement: Multimedia Appendix 1 [file ai-v4-e72987-s001.docx]

## **Multimedia Appendix 1. Link to Video Shown to Participants**

**Video link used for conditions:** <https://youtu.be/XCoX57su4Jw>

[
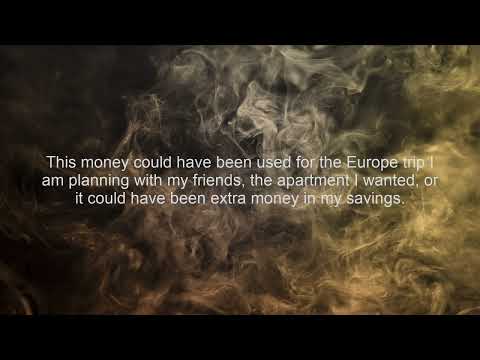
](https://youtu.be/XCoX57su4Jw)
